# Comparing Singlet Testing Schemes

**DOI:** 10.3390/e27050515

**Published:** 2025-05-11

**Authors:** George Cowperthwaite, Adrian Kent

**Affiliations:** 1 Centre for Quantum Information and Foundations, DAMTP, Centre for Mathematical Sciences, University of Cambridge, Wilberforce Road, Cambridge CB3 0WA, UK; 2 Perimeter Institute for Theoretical Physics, 31 Caroline Street North, Waterloo, ON N2L 2Y5, Canada

**Keywords:** bell non-locality, quantum cryptography, entanglement, state discrimination

## Abstract

We compare schemes for testing whether two parties share a two-qubit singlet state. The first, standard, scheme tests Braunstein–Caves (or CHSH) inequalities, comparing the correlations of local measurements drawn from a fixed finite set against the quantum predictions for a singlet. The second, alternative, scheme tests the correlations of local measurements, drawn randomly from the set of those that are θ-separated on the Bloch sphere, against the quantum predictions. We formulate each scheme as a hypothesis test and then evaluate the test power in a number of adversarial scenarios involving an eavesdropper altering or replacing the singlet qubits. We find the ‘random measurement’ test to be superior in most natural scenarios.

## 1. Introduction

Many quantum information protocols require two parties (Alice and Bob) to share a two-qubit singlet state:

(1) $$|\Psi^{-}\rangle =\frac{1}{\sqrt{2}}(|01\rangle -|10\rangle ),$$

where Alice holds the first qubit and Bob the second. Common examples of such two-party protocols include teleportation [1], summoning tasks [2,3,4,5] and other forms of distributed quantum computing (e.g., [6]), entanglement-based key distribution protocols (e.g., [7]), communication and information processing between collaborating agents in some protocols for position verification and position-based cryptography (e.g., [8,9,10]), and in relativistic quantum bit commitment (e.g., [11]).

The two parties should be confident they share singlets, both to ensure the protocol will execute as intended and to preclude the possibility of an adversarial third-party having interfered with the system for their own advantage. A natural method for distinguishing singlets from other quantum states is to measure a quantity for which the singlet attains a unique maximum. Common examples are the expressions in the CHSH [12] and Braunstein–Caves [13] inequalities. These have the additional advantage that they test Bell nonlocality. They can thus detect any adversarial attack that replaces the singlet qubits with classical physical systems programmed to produce deterministic or probabilistic results in response to measurements, since these can be modelled by local hidden variables. In this paper, we examine a quantity that has the same properties but has not previously been studied as a singlet test, the (anti-)correlation of outcomes of random measurements separated by a fixed angle θ∈(0,π/3), and compare it to schemes derived from the CHSH and Braunstein–Caves inequalities (e.g., [14,15,16,17]).

The singlet testing schemes we examine in this paper only require both parties to accurately perform projective measurements. Unlike singlet purification schemes [18], they do not need quantum computers or quantum memory. This makes them potentially advantageous when users’ technology is limited, or more generally when single-qubit measurements are cheaper compared to multi-qubit operations.

We assume that Alice and Bob are separated, and the singlet is created either by Alice or by a source separate from both Alice and Bob, before each qubit is transmitted to the respective party. An adversarial third party may intercept the qubits during transmission and alter them, either to obtain information or to disrupt the protocol. We refer to her as ‘Eve’, but emphasize that we are interested in protocols beyond key distribution and that her potential interference need not necessarily involve eavesdropping.

We compare the power of our proposed singlet testing schemes against four commonly studied attacks. These do not represent the full range of possible adversarial action, but illustrate why random measurement testing schemes can be advantageous in a variety of scenarios:

1. Single-qubit intercept–resend attack: Eve intercepts Bob’s qubit, performs a local projective measurement, notes the outcome, and sends the post-measurement state on to Bob. This could occur in a setting where Alice creates the singlet and transmits a qubit to Bob.
2. Bipartite state transformation: Eve intercepts both qubits and performs a quantum operation on them, replacing the singlet with some other two-qubit state that is sent to Alice and Bob.
3. LHV replacement: Eve replaces the singlet with a non-quantum system chosen so that Alice’s and Bob’s measurement outcomes are determined by local hidden variables instead of quantum entanglement.
4. Noisy quantum channel: This is described by a physically natural noise model (and is hence a special case of scenario 2, if we consider the noise as being due to Eve). This alters the singlet state as it is transmitted to Alice and Bob.

The advantages of these various attacks for Eve, in disrupting or obtaining information from the protocol, will depend on the context. We assume each offers Eve some potential advantage and focus on the extent to which Alice and Bob can detect the attacks.

We consider two different types of scheme that Alice and Bob may use to test the purported singlet:

- Braunstein–Caves test: Testing the Braunstein–Caves inequality [13] with a specific set of *N* measurement choices for which the singlet uniquely induces the maximum violation [19]. We often particularly focus on the N=2 case, the CHSH inequality [12], for which self-testing schemes have been extensively studied (e.g., [14,15,16,17]).
- Random measurement test: Alice and Bob choose random local projective measurements that are constrained to have a fixed separation angle on the Bloch sphere [20] and calculate the anti-correlation of their measurement outcomes. For a wide range of angles, this is uniquely maximized by the singlet.

The intuition, which we test and quantify, is that the random measurement test may generally be more efficient than Braunstein–Caves, as it tests anti-correlations for the same set of axis separations (π/2N), but chooses axes randomly over the Bloch sphere, providing Eve with less information about the test measurements, and hence offering her less scope to tailor her attack to minimize its detectability. In particular, the random measurement test is rotationally symmetric, and is hence sensitive to any attack by Eve that breaks rotational symmetry. It can also be applied to any θ, not just the discrete set of the form {π/2N:N∈N,N>1}.

We first describe these schemes and analyse their efficiency. We discuss their feasibility in the final section.

## 2. Materials and Methods

### 2.1. Braunstein–Caves Singlet Test

#### 2.1.1. Preliminaries

Alice and Bob wish to test whether they share a two-qubit singlet. It is possible that they instead share a not necessarily quantum system governed by a local hidden variable theory (see Section 3.3) or a more complex quantum system with further degrees of freedom. However, we start by assuming the parties are confident they share a (potentially mixed) two-qubit state $\rho_{AB}$. To start the test, Alice and Bob use an authenticated channel to fix a parameter N≥2 and uniformly randomly generate a projective measurement pair (A,B) from the set

(2) $${\left\{\left(a_{k},b_{k}\right)\right\}}_{k=0}^{N-1}\cup {\left\{\left(a_{k+1},b_{k}\right)\right\}}_{k=0}^{N-2}\cup \left\{\left(a_{0},b_{N-1}\right)\right\},$$

where

(3) $$\begin{array}{c} a_{k}=\left\{|m_{k\pi /2N}\rangle ,|m_{k\pi /2N+\pi /2}\rangle \right\}, \end{array}$$

(4) $$\begin{array}{c} b_{k}=\left\{|m_{(2k+1)\pi /4N}\rangle ,|m_{(2k+1)\pi /4N+\pi /2}\rangle \right\}, \end{array}$$

with $|m_{\theta }\rangle =\text{cos}\theta |0\rangle +\text{sin}\theta |1\rangle$; outcomes are labelled {1,−1}, respectively.

Next, Alice and Bob perform measurements *A* and *B*, respectively, on their qubit and store their outcomes as $O_{A}$ and $O_{B}$, respectively. They compute the following quantity:

(5) $$\hat{C}=\left\{\begin{array}{cc} -O_{A}O_{B} & \;\mathrm{when}\;\left(A,B\right)=\left(a_{0},b_{N-1}\right)\\ O_{A}O_{B} & \;\mathrm{otherwise} \end{array}\right.$$

through an authenticated classical channel. We call $\hat{C}$ the ‘Braunstein–Caves sample’, as it possesses properties derived from the Braunstein–Caves inequality [13].

$\hat{C}$ takes outcomes {1,−1}, so it follows a shifted Bernoulli distribution. The expected value of $\hat{C}$ resulting from a uniformly random choice of measurement bases (A,B) and the application of those measurements is as follows:

(6) $$E\left[\hat{C}\right]=\frac{1}{2N}\left(\sum_{k=0}^{N-1}E\left[a_{k},b_{k}\right]+\sum_{k=0}^{N-2}E\left[a_{k+1},b_{k}\right]-E\left[a_{0},b_{N-1}\right]\right),$$

with E[x,y] defined as the expected correlation between Alice and Bob’s measurement outcomes for choices (A,B)=(x,y). The expectation is bounded [19] for quantum states by

(7) $$|E\left[\hat{C}\right]|\leq \text{cos}\left(\frac{\pi }{2N}\right)$$

and the singlet saturates this bound [19].

As we review below, for the measurement choices defined in (3) and (4), the singlet *uniquely* achieves the minimum expectation (−cos(π/2N)). Any other state thus produces a detectable deviation in the sample mean of $\hat{C}$, given a large enough sample size, assuming perfect measurements.

#### 2.1.2. Calculating $E[\hat{C}]$ for $\rho_{AB}$

If Alice and Bob each utilise local projective measurements, a general combined measurement basis for a Braunstein–Caves test can be described by the following:

(8) $$\begin{array}{c} \{|m_{\theta }\rangle |m_{\phi }\rangle ,|m_{\theta +\frac{\pi }{2}}\rangle |m_{\phi +\frac{\pi }{2}}\rangle ,\\ \;|m_{\theta }\rangle |m_{\phi +\frac{\pi }{2}}\rangle ,|m_{\theta +\frac{\pi }{2}}\rangle |m_{\phi }\rangle \} \end{array}$$

where $|m_{\theta }\rangle =\text{cos}\theta |0\rangle +\text{sin}\theta |1\rangle$, with the first two results corresponding to the correlated outcomes and the final two results corresponding to the anticorrelated outcomes.

Define $a_{ijpq}=\langle ij|\rho_{AB}|pq\rangle$ for i,j,p,q∈{0,1}. The expected correlation between the outcomes of measurement $\{|m_{\theta }\rangle ,|m_{\theta +\frac{\pi }{2}}\rangle \}$ on Alice’s qubit and $\{|m_{\phi }\rangle ,|m_{\phi +\frac{\pi }{2}}\rangle \}$ on Bob’s qubit is given by the following:

(9) E{|mθ〉,|mθ+π2〉},{|mϕ〉,|mϕ+π2〉}=P(outcomessame)−P(outcomesdiffer)=2P(outcomessame)−1=2〈mϕ|〈mθ|ρAB|mθ〉|mϕ〉|2+2〈mϕ+π2|〈mθ+π2|ρAB|mθ+π2〉|mϕ+π2〉|2−1=cos2θcos2ϕ(a0000+a1111−a0101−a1010)+2cos2θsin2ϕ·Re(a0001−a1011)+2sin2θcos2ϕ·Re(a0010−a0111)+2sin2θsin2ϕ·Re(a0011+a0110).

Thus, by utilising values of θ,ϕ corresponding to the measurements in (3) and (4), quantity (6) can be evaluated as follows:

(10) E[C^]=12N∑k=0N−1E[ak,bk]+∑k=0N−2E[ak+1,bk]−E[a0,bN−1]=12cosπ2N(a0000+a1111−a0101−a1010)+cosπ2NRe(a0011+a0110)=cosπ2N〈Φ+|ρAB|Φ+〉−〈Ψ−|ρAB|Ψ−〉,

where $|\Phi^{+}\rangle =\frac{1}{\sqrt{2}}(|00\rangle +\left|11\rangle \right)$ and $|\Psi^{-}\rangle =\frac{1}{\sqrt{2}}(|01\rangle -\left|10\rangle \right)$.

Clearly, the minimum of $E[\hat{C}]$ is uniquely attained by the singlet.

### 2.2. Random Measurement Singlet Test

#### 2.2.1. Preliminaries

Alice and Bob wish to test whether they share a two-qubit singlet. Again, we start by assuming the parties are confident they share a (potentially mixed) two-qubit state $\rho_{AB}$. Alice uniformly randomly generates a projective qubit measurement

(11) $$\{|\psi_{A}\rangle ,|\psi_{A}^{⊥}\rangle \},$$

corresponding to outcomes {1,−1} and, likewise, Bob uniformly randomly generates a projective qubit measurement

(12) $$\{|\psi_{B}\rangle ,|\psi_{B}^{⊥}\rangle \},$$

from a set with the defining restriction that $|\psi_{A}\rangle$ and $|\psi_{B}\rangle$ must be separated by angle θ∈[0,π/2] on the Bloch sphere, so that $|\langle \psi_{A}|\psi_{B}\rangle |=\text{cos}\theta$. This separation can be achieved in many ways: for example, Alice and Bob could share a list of pre-agreed measurements or Bob could delay his measurement choice until Alice has made and communicated hers. The optimal method of achieving this depends on the parent protocol within which the shared singlet is required. For example, the pre-sharing of measurement choices may be reasonable when verifying singlets for use in a teleportation protocol, but may not be used in a key generation scheme, as a one-time pad could instead be pre-shared with similar resources.

Next, Alice and Bob perform their chosen measurements on their qubit and compute the product of their outcomes through an authenticated classical channel to obtain

(13) $$\hat{O}=O_{A}O_{B},$$

where $O_{A}$ and $O_{B}$ are the outcomes of Alice and Bob’s measurements, respectively. We call $\hat{O}$ the ‘random measurement sample’.

$\hat{O}$ takes outcomes {1,−1}, so it follows a shifted Bernoulli distribution. The expected value of $\hat{O}$ resulting from a uniformly random choice of θ separated measurement bases on the Bloch sphere and the application of those measurements is denoted by $E[\hat{O}]$.

It will be shown that, for this test, for 0≤θ<π/2 the singlet uniquely (among quantum states) attains the minimum expectation −cosθ. Any other state would produce a significant deviation in the sample mean of $\hat{O}$ given a large enough sample size. If Alice and Bob can implement a random measurement test precisely, for any given θ in the range, they can thus distinguish a source of singlets from a source of any other quantum state. Note, however, that since the measurements need to be individually calibrated, this is not a device-independent singlet test.

#### 2.2.2. Relation Between $|\psi_{A}\rangle$ and $|\psi_{B}\rangle$

If $|\psi_{A}\rangle$ and $|\psi_{B}\rangle$ are separated by angle θ on the Bloch sphere, they are related by

(14) $$\begin{array}{cc} |\psi_{B}\rangle = & U_{|\psi_{A}\rangle }P\left(\alpha \right)R\left(\theta /2\right)|0\rangle ,\\ |\psi_{B}^{⊥}\rangle = & U_{|\psi_{A}\rangle }P\left(\alpha \right)R\left(\theta /2\right)|1\rangle , \end{array}$$

for some α∈[0,2π), where $U_{|\psi_{A}\rangle }=|\psi_{A}\rangle \langle 0|+|\psi_{A}^{⊥}\rangle \langle 1|$ is a unitary transformation and

(15) $$P\left(\alpha \right)=\left(\begin{array}{cc} 1 & 0\\ 0 & e^{i\alpha } \end{array}\right),\;\;\;\;\;R\left(\theta /2\right)=\left(\begin{array}{cc} \text{cos}(\theta /2) & -\text{sin}(\theta /2)\\ \text{sin}(\theta /2) & \text{cos}(\theta /2) \end{array}\right),$$

in the computational basis. Thus,

(16) $$\begin{array}{cc} |\psi_{B}\rangle = & \langle 0|P\left(\alpha \right)R\left(\theta /2\right)|0\rangle |\psi_{A}\rangle +\langle 1|P\left(\alpha \right)R\left(\theta /2\right)|0\rangle |\psi_{A}^{⊥}\rangle ,\\ |\psi_{B}^{⊥}\rangle = & \langle 0|P\left(\alpha \right)R\left(\theta /2\right)|1\rangle |\psi_{A}\rangle +\langle 1|P\left(\alpha \right)R\left(\theta /2\right)|1\rangle |\psi_{A}^{⊥}\rangle . \end{array}$$

#### 2.2.3. $E_{AB}\left[\hat{O}\right]$ for Fixed Pair of Measurements

For ease of notation, define the following product states:

(17) $$\begin{array}{cc} |\psi_{AA}\rangle & =|\psi_{A}\rangle |\psi_{A}\rangle \;\;\;\;|\psi_{AA}^{⊥⊥}\rangle =|\psi_{A}^{⊥}\rangle |\psi_{A}^{⊥}\rangle ,\\ |\psi_{A^{⊥}A}\rangle & =|\psi_{A}^{⊥}\rangle |\psi_{A}\rangle \;\;\;\;|\psi_{AA^{⊥}}\rangle =|\psi_{A}\rangle |\psi_{A}^{⊥}\rangle ,\\ |\psi_{AB}\rangle & =|\psi_{A}\rangle |\psi_{B}\rangle \;\;\;\;|\psi_{AB}^{⊥⊥}\rangle =|\psi_{A}^{⊥}\rangle |\psi_{B}^{⊥}\rangle . \end{array}$$

Let $E_{AB}\left[\hat{O}\right]$ be the expected measurement correlation for a fixed choice of $|\psi_{A}\rangle$ and $|\psi_{B}\rangle$. Then,

(18) $$\begin{array}{cc} E_{AB}\left[\hat{O}\right] & =P\left[\hat{O}=1\right]-P\left[\hat{O}=-1\right]\\ \; & =2P[\hat{O}=1]-1\\ \; & =2\langle \psi_{AB}|\rho_{AB}|\psi_{AB}\rangle +2\langle \psi_{AB}^{⊥⊥}|\rho_{AB}|\psi_{AB}^{⊥⊥}\rangle -1. \end{array}$$

Using the expressions for $|\psi_{B}\rangle$ in (16) to evaluate each term individually,

(19) EAB[O^]=2cos2(θ/2)〈ψAA|ρAB|ψAA〉+2sin2(θ/2)〈ψAA⊥|ρAB|ψAA⊥〉+2Re[eiαsin(θ)〈ψAA|ρAB|ψAA⊥〉]+2cos2(θ/2)〈ψAA⊥⊥|ρAB|ψAA⊥⊥〉+2sin2(θ/2)〈ψA⊥A|ρAB|ψA⊥A〉−2Re[eiαsin(θ)〈ψAA⊥⊥|ρAB|ψA⊥A〉]−1.

#### 2.2.4. $E_{A}\left[\hat{O}\right]$ for a Fixed Alice Measurement

For a fixed measurement choice for Alice, the expected correlation $E_{A}\left[\hat{O}\right]$ among all Bob’s possible measurement choices is found by integrating over α in [0,π]. The integrals of $e^{i\alpha }$ and $e^{-i\alpha }$ vanish over this interval; hence,

(20) $$\begin{array}{cc} \; & E_{A}\left[\hat{O}\right]=\frac{1}{\pi }\int_{0}^{\pi }E_{AB}\left[\hat{O}\right]\;d\alpha \\ = & 2{\text{cos}}^{2}\left(\theta /2\right)\left[\langle \psi_{AA}|\rho_{AB}|\psi_{AA}\rangle +\langle \psi_{AA}^{⊥⊥}|\rho_{AB}|\psi_{AA}^{⊥⊥}\rangle \right]-1\\ \; & +2{\text{sin}}^{2}\left(\theta /2\right)\left[\langle \psi_{AA^{⊥}}|\rho_{AB}|\psi_{AA^{⊥}}\rangle +\langle \psi_{A^{⊥}A}|\rho_{AB}|\psi_{A^{⊥}A}\rangle \right]\\ = & \text{cos}\theta \left[2\langle \psi_{AA}|\rho_{AB}|\psi_{AA}\rangle +2\langle \psi_{AA}^{⊥⊥}|\rho_{AB}|\psi_{AA}^{⊥⊥}\rangle -1\right]. \end{array}$$

Note that $|\psi_{A}\rangle$ can be written as

(21) $$\begin{array}{cc} |\psi_{A}\rangle = & \text{cos}\left(\omega /2\right)|0\rangle +e^{i\beta }\text{sin}\left(\omega /2\right)|1\rangle ,\\ |\psi_{A}^{⊥}\rangle = & \text{sin}\left(\omega /2\right)|0\rangle -e^{i\beta }\text{cos}\left(\omega /2\right)|1\rangle , \end{array}$$

for some ω∈[0,π] and β∈[0,2π), so that

(22) $$\begin{array}{cc} |\psi_{AA}\rangle = & \frac{1}{2}\left(1+\text{cos}\omega \right)|00\rangle +\frac{1}{2}e^{i\beta }\text{sin}\omega |01\rangle \\ \; & +\frac{1}{2}e^{i\beta }\text{sin}\omega |10\rangle +\frac{1}{2}e^{2i\beta }\left(1-\text{cos}\omega \right)|11\rangle ,\\ |\psi_{AA}^{⊥⊥}\rangle = & \frac{1}{2}\left(1-\text{cos}\omega \right)|00\rangle -\frac{1}{2}e^{i\beta }\text{sin}\omega |01\rangle \\ \; & -\frac{1}{2}e^{i\beta }\text{sin}\omega |10\rangle +\frac{1}{2}e^{2i\beta }\left(1+\text{cos}\omega \right)|11\rangle . \end{array}$$

For ease of notation, define the following quantities:

(23) $$a_{ijpq}=\langle ij|\rho_{AB}|pq\rangle ,$$

for i,j,p,q∈{0,1}. Using these quantities, Equation (20) provides the following:

(24) EA[O^]=cosθ2〈ψAA|ρAB|ψAA〉+2〈ψAA⊥⊥|ρAB|ψAA⊥⊥〉−1=cosθ[12(1+cosω)2a0000+12(1−cosω)2a1111+12(1−cosω)2a0000+12(1+cosω)2a1111+sin2ω·a0101+sin2ω·a1010+2sin2ω·Re[a0110]−1+f(eiβ,e2iβ,e−iβ,e−2iβ)]=cosθ[cos2ω−(cos2ω+1)(a0101+a1010)+2sin2ω·Re[a0110]+f(eiβ,e2iβ,e−iβ,e−2iβ)],

where *f* is a function representing a linear combination of its arguments.

#### 2.2.5. Calculating $E[\hat{O}]$

The expected correlation over all Alice’s possible measurement choices is found by integrating (24) over β in [0,2π] and over ω in [0,π], with the Jacobian sinω appropriate for integration over a sphere surface. The integral of $e^{in\beta }$ vanishes over the interval [0,2π]; hence,

(25) E[O^]=14π∫02π∫0πEA[O^]sinωdωdβ=12cosθ∫0π[cos2ω−(cos2ω+1)(a0101+a1010)+2sin2ω·Re[a0110]]sinωdω=cosθ(13−23(a0101+a1010)+43Re[a0110])=cosθ(13−43〈Ψ−|ρAB|Ψ−〉),

where $\langle \Psi^{-}|\rho_{AB}|\Psi^{-}\rangle$ is the fidelity between $\rho_{AB}$ and the singlet state.

Clearly, the minimum of $E[\hat{O}]$ is uniquely attained by the singlet.

## 3. Results

We will now describe and compare hypothesis tests for the singlet using (i) the Braunstein–Caves samples with parameter N or (ii) random measurement samples with θ=π/2N. We link our choice of θ to *N* in this way, as this ensures both tests induce equal expected correlations when measuring singlets, allowing for a clear comparison of the effect of deviations. We recall that the test samples both follow shifted Bernoulli distributions:

(26) $$\begin{array}{cc} \mathrm{BC}\;\mathrm{sample}\;\hat{C} & ∼2\cdot \mathrm{Bernoulli}(p)-1,\\ \mathrm{RM}\;\mathrm{sample}\;\hat{O} & ∼2\cdot \mathrm{Bernoulli}(q)-1, \end{array}$$

with parameters defined through the expectation values in (10) and (25) as

(27) $$\begin{array}{cc} p & =\frac{1}{2}+\frac{1}{2}\text{cos}\left(\frac{\pi }{2N}\right)\left(\langle \Phi^{+}|\rho_{AB}|\Phi^{+}\rangle -\langle \Psi^{-}|\rho_{AB}|\Psi^{-}\rangle \right),\\ q & =\frac{1}{2}+\frac{1}{2}\text{cos}\left(\frac{\pi }{2N}\right)\left(\frac{1}{3}-\frac{4}{3}\langle \Psi^{-}|\rho_{AB}|\Psi^{-}\rangle \right). \end{array}$$

If we denote the sample mean of *n* Braunstein–Caves samples as $\bar{C}$ and the sample mean of *n* random measurement samples as $\bar{O}$, then they both follow shifted binomial distributions:

(28) $$\begin{array}{cc} \mathrm{BC}\;\mathrm{sample}\;\mathrm{mean}\;\bar{C} & ∼\frac{2}{n}B\left(n,p\right)-1,\\ \mathrm{RM}\;\mathrm{sample}\;\mathrm{mean}\;\bar{O} & ∼\frac{2}{n}B\left(n,q\right)-1. \end{array}$$

### 3.1. Description of Hypothesis Tests

We aim to test the following hypotheses:

(29) $$H_{0}:\rho_{AB}=|\Psi^{-}\rangle \langle \Psi^{-}|\;\;\;v\;\;\;H_{1}:\rho_{AB}\neq |\Psi^{-}\rangle \langle \Psi^{-}|.$$

If we wish to conduct the test using Braunstein–Caves samples, we generate *n* samples of $\hat{C}$ (as in Section 2.1) and let the test statistic be $\bar{C}$.

If we wish to conduct the test using random measurement samples, we generate *n* samples of $\hat{O}$ (as in Section 2.2) and let the test statistic be $\bar{O}$.

Let α be the desired size of the test, defined as the probability the null hypothesis is erroneously rejected when Alice and Bob do in fact share a singlet. The critical region *R* is a set of values for the test statistic for which the null hypothesis is rejected. For both tests, we wish to define *R* as follows:

(30) $$R=\left\{x:x>\frac{2z_{\alpha ,n}}{n}-1\right\},$$

where $z_{\alpha ,n}$ is defined as the upper α-quantile of a $B\left(n,\frac{1}{2}-\frac{1}{2}\text{cos}\left(\frac{\pi }{2N}\right)\right)$ distribution. However, binomial quantiles can only take discrete values, so we are often unable to select one exactly correponding to α. To rectify this, we instead set $z_{\alpha ,n}$ to be the smallest integer, such that $P\left(X>z_{\alpha ,n}|X∼B\left(n,\frac{1}{2}-\frac{1}{2}\text{cos}\left(\frac{\pi }{2N}\right)\right)\right)<\alpha$ and extend *R* to a critical decision region $R^{+}$, where if our test statistic exactly equals $2z_{\alpha ,n}/n-1$, we decide to reject the null hypothesis with probability *q*, where *q* is chosen so that $P\left(X\in R^{+}|X∼B\left(n,\frac{1}{2}-\frac{1}{2}\text{cos}\left(\frac{\pi }{2N}\right)\right)\right)=\alpha$.

Through (28), it follows that $P\left(\bar{C}\in R^{+}|H_{0}\right)=P\left(\bar{O}\in R^{+}|H_{0}\right)=\alpha$, as $\bar{C}$ and $\bar{O}$ are identically distributed under the null hypothesis.

The power functions $\pi_{BC}$ and $\pi_{RM}$ for each test describe the probability the null hypothesis is rejected given the density matrix of the state being tested, and are defined using (27)–(30) as follows:

(31) $$\begin{array}{cc} \pi_{BC}\left(\rho_{AB}\right) & =P(X\in R^{+}|X∼B\left(n,p\right)),\\ \pi_{RM}\left(\rho_{AB}\right) & =P(X\in R^{+}|X∼B\left(n,q\right)). \end{array}$$

It is clear that

(32) $$\begin{array}{cc} \pi_{BC}\left(\rho_{AB}\right)>\pi_{RM}\left(\rho_{AB}\right) & \Leftrightarrow p>q\\ & \Leftrightarrow E\left[\hat{C}\right]>E\left[\hat{O}\right], \end{array}$$

so whether (i) or (ii) is better at detecting non-singlet states in a given scenario can be determined by comparing the values of $E[\hat{C}]$ and $E[\hat{O}]$ associated with the testing of typical states arising from that scenario.

For large *n*, the asymptotic power functions are described by the central limit theorem. If Φ is the cumulative distribution function of a N(0,1) distribution, then, as n→∞, the following holds:

(33) $$\begin{array}{cc} \pi_{BC}\left(\rho_{AB}\right) & ∼1-\Phi \left(\frac{{\tilde{z}}_{\alpha ,n}+0.5-np}{\sqrt{np(1-p)}}\right),\\ \pi_{RM}\left(\rho_{AB}\right) & ∼1-\Phi \left(\frac{{\tilde{z}}_{\alpha ,n}+0.5-nq}{\sqrt{nq(1-q)}}\right), \end{array}$$

where the ‘+0.5’ terms are the appropriate correction for a continuous limit of a discrete distribution and ${\tilde{z}}_{\alpha ,n}$ is the upper α-quantile of a $N(np_{0},np_{0}\left(1-p_{0}\right))$ distribution, with $p_{0}=\frac{1}{2}-\frac{1}{2}\text{cos}\left(\frac{\pi }{2N}\right)$.

### 3.2. Comparison for Simple Intercept-Resend Attack

Consider the scenario in which Eve manages to intercept Bob’s qubit and performs the following measurement:

(34) $$\{|\alpha \rangle =\text{cos}\psi |0\rangle +e^{i\beta }\text{sin}\psi |1\rangle ,|\alpha^{⊥}\rangle \},$$

before sending the post-measurement qubit on to Bob. As the singlet can be expressed as follows:

(35) $$|\Psi^{-}\rangle =\frac{1}{\sqrt{2}}\left|\alpha \rangle \right|\alpha^{⊥}\rangle -\frac{1}{\sqrt{2}}|\alpha^{⊥}\rangle |\alpha \rangle ,$$

it is clear that the post-measurement state will be the following:

(36) $$\rho_{AB}=\left\{\begin{array}{cc} \left|\alpha \rangle \langle \alpha |⊗\right|\alpha^{⊥}\rangle \langle \alpha^{⊥}| & \mathrm{with}\;\mathrm{probability}\;1/2\\ |\alpha^{⊥}\rangle \langle \alpha^{⊥}\left|⊗|\alpha \rangle \langle \alpha \right| & \mathrm{with}\;\mathrm{probability}\;1/2. \end{array}\right.$$

The expected test samples are independent of Eve’s measurement outcome and are calculated in Table 1 using (10) and (25).

These results show that a single-qubit intercept–resend attack reduces $E[\hat{O}]$ to 1/3 of its singlet value, and reduces $E[\hat{C}]$ to between 1/4 and 1/2 of its singlet value, depending on the measurement made by Eve.

**Figure 1:**
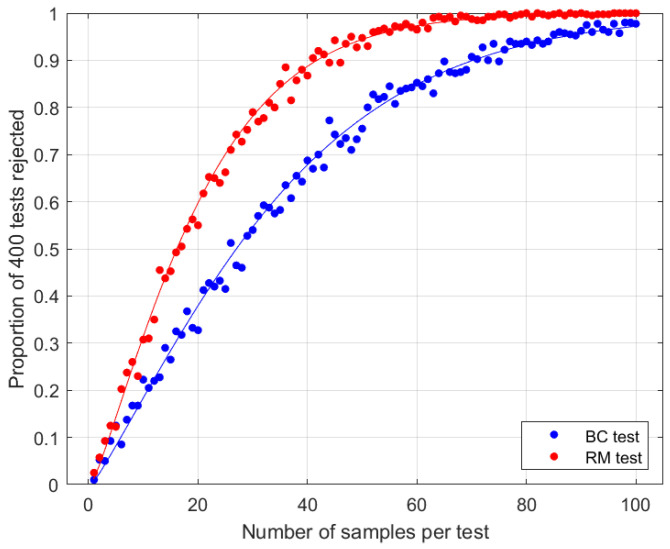
Results when Eve measures singlets in the computational basis and transmits the outcome states (see Section 3.2). Test parameters are N=2 and θ=π/4. Curves are theoretical asymptotic power functions (33). Dots are empirical data, representing the proportion of 400 simulated tests leading to a rejection of the null hypothesis at the 1% level.

Eve will choose (ψ,β) to achieve a desired balance of minimal disruption and maximal information gain; hence, her choice will depend on the parent protocol within which Alice and Bob intended to use the singlet. For example, if BB84 is the parent protocol, it is known [21] that the Breidbart basis (β=0,ψ=π/8) is optimal for Eve; hence, $E\left[\hat{O}\right]>E\left[\hat{C}\right]$ and the measurement test has greater power. More generally, if Eve’s priority is to choose a basis which minimises disruption, the random measurement test will be more powerful (see Figure 1).

It is clear that choosing *N* to be as large as possible will maximise the difference between the expected correlations under the null and alternative hypotheses, leading to a more powerful test for both schemes. This implies that choosing θ=0 is optimal for the random measurement test in this scenario; hence, it is optimal for Alice and Bob to use the same randomly chosen measurements if they know they are testing a singlet and a post-measurement state. However, it is known [22] (see Section 3.4) that for θ=0, the test does not distinguish the singlet from a class of simple LHV models.

### 3.3. Comparison for Bipartite State Transformation Attack

Consider the scenario in which Eve intercepts both qubits and manipulates them so that the singlet is transformed into some other state $\rho_{AB}$, with the following singlet fidelity:

(37) $$\langle \Psi^{-}|\rho_{AB}|\Psi^{-}\rangle =1-\epsilon .$$

While we permit any ϵ∈[0,1], we are particularly interested in small values. The expected test samples are calculated in Table 2 using (10) and (25).

The results show that $E[\hat{O}]$ increases with ϵ at linear rate 4cos(π/2N)/3, while $E[\hat{C}]$ increases with $\left(\epsilon +\langle \Phi^{+}|\rho_{AB}|\Phi^{+}\rangle \right)$ at linear rate cos(π/2N); thus, the test of greater power can be identified by comparing the values of $\langle \Phi^{+}|\rho_{AB}|\Phi^{+}\rangle$ and ϵ. Note that the orthogonality of Bell states imposes the constraint $0\leq \langle \Phi^{+}|\rho_{AB}|\Phi^{+}\rangle \leq \epsilon$.

When $0\leq \langle \Phi^{+}|\rho_{AB}|\Phi^{+}\rangle <\epsilon /3$, we have $E\left[\hat{O}\right]>E\left[\hat{C}\right]$, and when $\epsilon /3<\langle \Phi^{+}|\rho_{AB}|\Phi^{+}\rangle \leq \epsilon$, we have $E\left[\hat{C}\right]>E\left[\hat{O}\right]$, while for $\langle \Phi^{+}|\rho_{AB}|\Phi^{+}\rangle =\epsilon /3$, both tests are equally strong.

**Figure 2:**
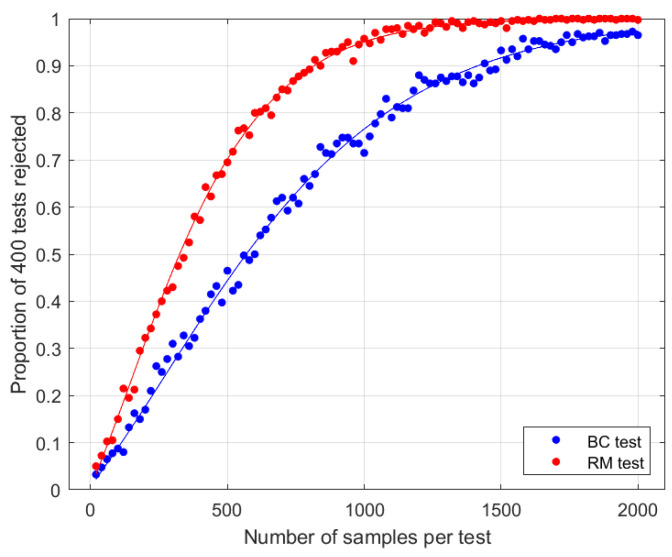
Results when Eve transforms singlets to states with $\langle \Psi^{-}|\rho_{AB}|\Psi^{-}\rangle =0.9$ and with $\langle \Phi^{+}|\rho_{AB}|\Phi^{+}\rangle =0$, choosing the latter to be undetectable as possible with the BC test for the given fidelity (see Section 3.3). Test parameters are N=2 and θ=π/4. Curves are theoretical asymptotic power functions (33). Dots are empirical data, representing the proportion of 400 simulated tests leading to a rejection of the null hypothesis at the 1% level.

As an example, in a scenario where Eve prioritises being as undetectable as possible for a given ϵ, she would choose a transformation with $\langle \Phi^{+}|\rho_{AB}|\Phi^{+}\rangle =0$, so the random measurement test would be superior in this case (see Figure 2).

One way to overcome the uncertainty in the value of $\langle \Phi^{+}|\rho_{AB}|\Phi^{+}\rangle$ is to require Alice and Bob to apply the same randomly chosen unitary operation *U* to both of their qubits before measurement, without remembering the identity of *U*. This effectively transforms their shared system to a mixed state of the following form:

(38) $$\left(1-\epsilon \right)|\Psi^{-}\rangle \langle \Psi^{-}|+\frac{\epsilon }{3}\left(I-|\Psi^{-}\rangle \langle \Psi^{-}|\right)$$

as the singlet component remains invarient under a U⊗U transformation, while the complement becomes maximally mixed. This ensures that both tests are equivalently strong when testing the resulting state, as $\langle \Phi^{+}|\rho_{AB}|\Phi^{+}\rangle =\epsilon /3$.

The largest possible choice of parameter *N* leads to the test of greater power for each type of scheme, much as it did in Section 3.2.

### 3.4. Comparison for LHV Replacement Attack

Consider the scenario in which Eve intercepts both qubits and replaces them with a not necessarily quantum system where the correlation between Alice and Bob is governed entirely by a local hidden variable (LHV) theory. Using the Braunstein–Caves inequality [13], the expected value of the Braunstein–Caves sample is bounded for integers N≥2 as

(39) $$|E\left[\hat{C}\right]|\;\leq 1-\frac{1}{N}<\text{cos}\left(\frac{\pi }{2N}\right),$$

for measurements of a two-sided LHV system. It is also known (Theorem 1 in [20]) that the expected value of the random measurement sample for θ=π/2N is bounded for integers N≥2 as follows:

(40) $$|E\left[\hat{O}\right]|\;\leq 1-\frac{1}{N}<\text{cos}\left(\frac{\pi }{2N}\right),$$

for measurements of a two-sided LHV system. The strictly positive difference between correlations resulting from LHV models and singlets implies that both tests can detect when the correlation between Alice and Bob’s measurement outcomes is caused by an LHV theory, with a power that is uniformly bounded for all possible LHV theories.

For N≥2, the optimal parameters for both types of scheme are found by selecting the value of *N* that maximises the difference between expected singlet correlations and the bound on LHV correlations. This difference is defined in (39) and (40) as follows:

(41) $$D\left(N\right)=\text{cos}\left(\frac{\pi }{2N}\right)+\frac{1}{N}-1.$$

As D(2)≈0.207, D(3)≈0.199 and $D^{\prime }\left(N\right)<0$ for N≥3, it follows that D(N) is maximised by N=2 over integer inputs greater than 1, providing an optimal minimum bound on test power for both schemes.

This result does not identify which value of θ leads to the random measurement test with the greatest power for detecting LHV models, as it is possible to use any θ∈[0,π/2], not just the discrete selection considered above, and the gap is not generally given by (41). This question was explored further in [23] and resolved numerically in [24]. The optimal value for detecting general LHV models that are optimized to simulate the singlet is θ=π/4; the optimal value for detecting general LHV models that are optimzed to simulate the singlet, with the constraint that they provide perfect anticorrelations for measurements about the same axis, is θ=π/5.

It is also interesting to compare the optimal value of θ for detecting the LHVs given by Bell’s original model [22], which is defined such that Alice’s measurement on one hemisphere of the Bloch sphere leads to outcome +1 and the other leads to outcome −1, with Bob’s measurement providing opposite values on the same hemispheres. For this model, it is easy to verify that

(42) $$E\left[\hat{O}\right]=-1+\frac{2\theta }{\pi },$$

with the difference between this expected correlation and that for the singlet being

(43) $$\tilde{D}\left(\theta \right)=\text{cos}\theta +\frac{2\theta }{\pi }-1.$$

**Figure 3:**
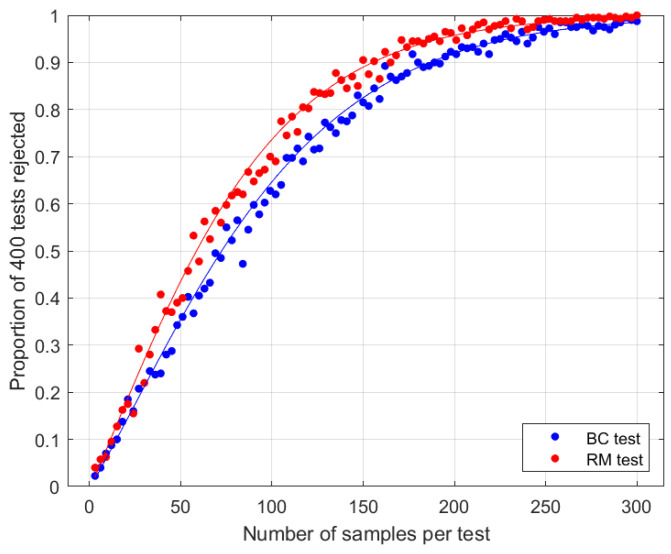
Results when Eve replaces singlets with Bell’s LHV model. Test parameters are N=2 and θ=arcsin(2/π), the optimal choice for the random measurement test in this case (see Section 3.4). Curves are theoretical asymptotic power functions (33). Dots are empirical data, representing the proportion of 400 simulated tests leading to a rejection of the null hypothesis at the 1% level.

This quantity is maximised by θ=arcsin(2/π)≈0.6901, leading to $\tilde{D}\left(\text{arcsin}\left(2/\pi \right)\right)\approx 0.2105$. Hence, the random measurement test with this parameter has the greatest power for detecting this class of LHV models (see Figure 3). For comparison, $\tilde{D}\left(\pi /4\right)\approx 0.2071$.

Since $\tilde{D}\left(\theta \right)>0$ for all θ∈(0,π/2), a test with any θ in this range would detect Bell’s LHV models with some efficiency.

### 3.5. Comments on LHV Model Testing with Measurement Errors

When Alice and Bob program their measurement devices during a test, there is a possibility they incur small calibration errors. These could be realised as small deviations in their measurement angles on the Bloch sphere. We fix δ>0 as a bound on the magnitude of a deviation in any single measurement for both Alice and Bob.

We examine the effect of such errors on the random measurement and Braunstein–Caves schemes.

#### 3.5.1. Random Measurement Scheme

Theorem 1 of Ref. [20] provides a bound on the expected value of a random measurement sample from an LHV model in this error regime. The theorem equivalently states that for any LHV model, any integer N≥2 and any $\theta \in \left[\pi /2N,\pi /2(N-1)\right)$, we have $-1+1/N\leq E[\hat{O}]\leq 1-1/N$.

For δ<π/8N(N−1), it follows that the expected value of a random measurement sample from any LHV model with chosen angle θ=π/2N+2δ satisfies

(44) $$|E\left[\hat{O}\right]|\;\leq 1-\frac{1}{N}.$$

In this setting, the greatest assured difference between the expected correlation for a singlet and that for an LHV model over all possible δ-bounded errors is

(45) $$D\left(N\right)=\text{cos}\left(\frac{\pi }{2N}+4\delta \right)-\left(1-\frac{1}{N}\right).$$

D(N) is positive when δ<(arccos(1−1/N)−π/2N)/4, implying that the random measurement test can distinguish between singlet and LHV models in the presence of δ-bounded measurement errors when δ<min{π/8N(N−1),(arccos(1−1/N)−π/2N)/4}.

For N≥3, this required bound on δ converges monotonically to 0 as *N* increases. This implies that the scheme can only reliably tolerate a smaller range of absolute measurement errors when *N* is large, suggesting that schemes with reasonably large θ may be more robust.

#### 3.5.2. Braunstein–Caves Scheme

The expected value of a Braunstein–Caves sample from an LHV model in this error regime is still bounded as

(46) $$|E\left[\hat{C}\right]|\;\leq 1-\frac{1}{N},$$

as the Braunstein–Caves inequality holds independently of Alice and Bob’s measurement choices.

In this setting, the expected correlation for a singlet over all δ-bounded measurement errors can be calculated using (6) and (9) by shifting the usual Braunstein–Caves measurement angles for Alice by $\epsilon_{A}$ and likewise for Bob by $\epsilon_{B}$, where both $\epsilon_{A}$ and $\epsilon_{B}$ represent δ-bounded errors, leading to the following:

(47) $$E\left[\hat{C}\right]=-\text{cos}\left(\frac{\pi }{2N}\right)\text{cos}\left(2\left(\epsilon_{A}-\epsilon_{B}\right)\right).$$

This implies that the greatest assured difference between the expected correlation for a singlet and that of an LHV model over all possible δ-bounded errors is

(48) $$D\left(N\right)=\text{cos}\left(\frac{\pi }{2N}\right)\text{cos}\left(4\delta \right)-\left(1-\frac{1}{N}\right).$$

D(N) is positive when δ<(arccos(1−1/N)−π/2N)/4, implying that, under this condition, the Braunstein–Caves test can distinguish between singlet and LHV models in the presence of δ-bounded measurement errors.

For N≥2, this required bound on δ converges monotonically to 0 as *N* increases. This implies that the scheme can only reliably tolerate a smaller range of absolute measurement errors when *N* is large, again suggesting that schemes with a small *N* may be more robust.

#### 3.5.3. Conclusions

In summary, it is shown that both schemes are still able to distinguish between singlet and LHV models in the presence of small deviations in the intended measurement angle. As *N* becomes large, we become less sure of the robustness of each scheme, as the proven range of tolerable measurement errors decreases.

### 3.6. Comparison for Noisy Quantum Channel

Consider the scenario in which Eve takes no action, but the quantum channel used for state transmission to Alice and Bob is affected by noise. Different quantum channels are afflicted with different types of noise; however, as a simple example, we can consider a depolarising channel that replaces the singlet with the maximally mixed state with probability δ.

The effect of this noise on a singlet can be modelled using two-qubit Werner states, with these being the only set of states that is invariant under arbitrary unitary transformations acting equally on both qubits [25].

The two-qubit Werner state can be defined as follows:

(49) $$W_{\delta }=\left(1-\delta \right)|\Phi^{-}\rangle \langle \Phi^{-}|+\frac{\delta }{4}I,$$

where δ parametrises the strength of the noise, with δ=0 corresponding to a pure singlet state in the absence of noise.

The expected test samples are calculated in Table 3 using (10) and (25).

Hence, both tests are equally powerful in testing for depolarising noise. As in Section 3.2 and Section 3.3, a larger value of *N* leads to a test of greater power, so the choice of a large *N* and θ=0 would be optimal.

As an additional example, we can consider the effect of a simple dephasing channel which acts on a qubit as Pauli gate *Z* with probability *p*. The effect of this noise on a singlet can be described as follows:

(50) $$\begin{array}{cc} \Delta_{p} & ={\left(1-p\right)}^{2}|\Phi^{-}\rangle \langle \Phi^{-}|\\ \; & +p\left(1-p\right)\left(I⊗Z\right)|\Phi^{-}\rangle \langle \Phi^{-}|\left(I⊗Z\right)\\ \; & +p\left(1-p\right)\left(Z⊗I\right)|\Phi^{-}\rangle \langle \Phi^{-}|\left(Z⊗I\right)\\ & +p^{2}\left(Z⊗Z\right)|\Phi^{-}\rangle \langle \Phi^{-}|\left(Z⊗Z\right)\\ \; & =\left({\left(1-p\right)}^{2}+p^{2}\right)|\Phi^{-}\rangle \langle \Phi^{-}\left|+2p\left(1-p\right)\right|\Phi^{+}\rangle \langle \Phi^{+}|, \end{array}$$

where we restrict 0<p<1.

The expected test samples are calculated in Table 4 using (10) and (25).

It is clear that the random measurement test has greater power in testing for this type of dephasing for any 0<p<1. Just as in Section 3.2 and Section 3.3, a larger value of *N* leads to a test of greater power, so a choice of large *N* and θ=0 would be optimal.

## 4. Discussion

While there is no universally superior choice of singlet test, we have seen that the random measurement test is theoretically superior or equal in many natural scenarios, including in the detection of intercept–resend or transformation attacks, where Eve prioritises minimising her chance of detection, distinguishing LHV models, and detecting rotationally invariant noise.

These results provide a rationale for considering the random measurement test for singlet verification over more conventional CHSH schemes (e.g., [14,15,16,17]). A complete analysis would consider the full range of attacks open to Eve and the full range of tests available for A and B. This would define a two-party game (with A and B collaborating as one party and Eve as the other), in which the optimal strategy for each party is likely probabilistic. However, Eve’s actions may be limited depending on how the singlets are generated and distributed and on the technologies available to her. Also, Alice and Bob may be able to exclude non-quantum LHV attacks if they can test qubits before measurement to ensure they are in the appropriate physical state.

Our discussion has mainly focussed on the ideal case, in which Alice and Bob can carry out perfectly precise measurements. Establishing that random measurement tests have an advantage in this case shows they are potentially valuable options, and motivates the development of technology that can implement them more easily and precisely. However, at present, imprecisions need to be taken into account when assessing the relative feasibility, advantages and costs of all the considered tests. For example, the Braunstein–Caves test only requires the calibration of measurement devices in a finite number (2N) of orientations around a great circle on the Bloch sphere, while the random measurement test requires the ability to measure all possible orientations. The Braunstein–Caves test may thus be a more desirable choice if calibrating detectors or, equivalently, if manipulating qubits precisely is difficult. An analysis of the feasibility of carrying out random measurement tests with current or foreseeable future technology—a task for future work—would illuminate these tradeoffs.

In principle, the random measurement protocol can be implemented in various ways, each of which requires some resources. One option is for Alice and Bob to pre-coordinate their measurements. This requires secure classical communication and/or secure classical memory, albeit not necessarily a large amount. For example, if Alice and Bob choose from a pre-agreed list of $10^{6}$ approximately uniformly distributed axes on the Bloch sphere, they can specify a measurement pair with about 40 bits, choosing pairs separated by the chosen θ to within error $≲3\times 10^{-4}$. Consuming secure classical communication and/or memory at this rate is not hugely demanding, and may be a reasonable option in many quantum cryptographic and communication scenarios. However, relatively precise pre-coordinated measurements effectively define (if pre-agreed) or consume (if securely communicated) large amounts of a shared secret key. Singlet verification may be required for only a small fraction of the shared singlets. Still, the advantage is, at best, context-dependent in protocols that aim to generate one-time pads.

An alternative, if Bob has short-term quantum memory, is for Alice to communicate her measurement choice after Bob receives and stores his qubit. Each can then define their measurement choice using locally generated or stored random bits, and Bob can delay his measurement choice until he receives Alice’s, with no additional security risk.

Another possible option is for Alice and Bob to choose measurements randomly and independently, and then sort their results into approximately θ-separated pairs post-measurement for some discrete set of θ in the range [0,π/2]. This effectively means carrying out random measurement tests for each θ in the chosen set, up to some chosen finite precision. This protocol effectively uses a random variable θ, and further analysis is needed to characterise its efficiency. The Braunstein–Caves protocol can be similarly adapted to avoid pre-coordination if Alice and Bob each independently choose measurements from set (2) and then sort their results into pairs that correspond to complete elements of (2). For a test with parameter *N*, they would, on average, retain a fraction 2/N of their samples. If the remainder are discarded, this requires them to multiply their initial sample size by N/2 to compensate. However, some of the discarded data could be used for further Braunstein–Caves tests if N is factorisable. Other anti-correlation tests could, in principle, be carried out on the remainder (although the finite precision loophole for measurements on the circle [23] needs to be allowed for). In the N=2 (CHSH) case, there is no loss of efficiency, as all choices by Alice and Bob would correspond to an allowed pair.

Larger values of *N* provide more powerful tests for detecting bipartite state transformation attacks and rotationally invariant noise, while the smallest possible *N* is optimal for detecting LHV correlations. Alice and Bob should thus either choose *N* according to which type of attack is most likely or—if they are in the type of game-theoretic scenario discussed above—act against the potential use of any of the attacks by employing a probabilistic strategy that mixes different values of *N*.

In the case N=2, there is a natural sense that the random measurement test is at least as good at, or better than, the Braunstein–Caves test in every scenario. In Section 3.2, Eve’s goal could be to carry out an intercept-resend with minimum probability of detection (i.e., ψ=0 or β=0), in which case the random measurement test is more powerful. In Section 3.3, Eve’s goal could be to carry out a state replacement that achieves fidelity 1−ϵ with minimum probability of detection (i.e., $\langle \Phi^{+}|\rho_{AB}|\Phi^{+}\rangle =0$), in which case the random measurement test is again more powerful. In Section 3.4 and Section 3.6, both tests are equally good for all variations. In Section 3.5, it is shown that both tests are still effective in the presence of small measurement calibration errors.

Our results thus make a clear case for the consideration of random measurement tests, and add motivation to continue work [23,24] focused on identifying their power for the full range of θ∈(0,π/3).

Random measurement tests are, at present, technologically challenging. More work is also needed to characterise their robustness in real-world applications where finite precision is inevitable, with various plausible error models, and where there may be a wide range of plausible adversarial attacks. For example, Eve might employ a mixture of the attacks discussed above, choosing different attacks randomly for different singlets, and/or combinations of these attacks on each singlet. That said, our results suggest that random measurement tests should be considered, as and when the technology allows, in scenarios where efficient singlet testing is critical and the costs of classical and/or quantum memory resources are relatively negligible. The optimal testing strategies against general attacks likely also involve random mixtures of tests. It would thus also be very interesting to explore the advantages of random measurement tests in more sophisticated testing strategies, such as mixtures of random measurements with different angles [24] and routed singlet tests [26] using random measurements.

## Figures and Tables

**Table 1:** Comparison of expectation values for post-measurement state and singlet, with the BC test using parameter *N* and the RM test using θ=π/2N.

	$\rho_{\mathrm{AB}}$	$|\Psi^{-}\rangle$
$E[\hat{C}]$	$\left(-\frac{1}{2}+\frac{1}{4}{\text{sin}}^{2}\left(2\psi \right){\text{sin}}^{2}\left(2\beta \right)\right)\text{cos}\left(\frac{\pi }{2N}\right)$	$-\text{cos}\left(\frac{\pi }{2N}\right)$
$E[\hat{O}]$	$-\frac{1}{3}\text{cos}\left(\frac{\pi }{2N}\right)$	$-\text{cos}\left(\frac{\pi }{2N}\right)$

**Table 2:** Comparison of expectation values for post-transformation state and singlet, with the BC test using parameter *N* and the RM test using θ=π/2N.

	$\rho_{\mathrm{AB}}$	$|\Psi^{-}\rangle \langle \Psi^{-}|$
$E[\hat{C}]$	$\left(-1+\epsilon +\langle \Phi^{+}|\rho_{AB}|\Phi^{+}\rangle \right)\text{cos}\left(\frac{\pi }{2N}\right)$	$-\text{cos}\left(\frac{\pi }{2N}\right)$
$E[\hat{O}]$	$\left(-1+\frac{4}{3}\epsilon \right)\text{cos}\left(\frac{\pi }{2N}\right)$	$-\text{cos}\left(\frac{\pi }{2N}\right)$

**Table 3:** Comparison of expectation values for the Werner state and singlet, with the BC test using parameter *N* and the RM test using θ=π/2N.

	$W_{\delta }$	$|\Psi^{-}\rangle \langle \Psi^{-}|$
$E[\hat{C}]$	$-\left(1-\delta \right)\text{cos}\left(\frac{\pi }{2N}\right)$	$-\text{cos}\left(\frac{\pi }{2N}\right)$
$E[\hat{O}]$	$-\left(1-\delta \right)\text{cos}\left(\frac{\pi }{2N}\right)$	$-\text{cos}\left(\frac{\pi }{2N}\right)$

**Table 4:** Comparison of expectation values for the dephased state and singlet, with the BC test using parameter *N* and the RM test using θ=π/2N.

	$\Delta_{p}$	$|\Psi^{-}\rangle \langle \Psi^{-}|$
$E[\hat{C}]$	$-\left(1-2p\left(1-p\right)\right)\text{cos}\left(\frac{\pi }{2N}\right)$	$-\text{cos}\left(\frac{\pi }{2N}\right)$
$E[\hat{O}]$	$-\left(1-\frac{8}{3}p\left(1-p\right)\right)\text{cos}\left(\frac{\pi }{2N}\right)$	$-\text{cos}\left(\frac{\pi }{2N}\right)$

Note the superior performance of the random measurement test due to its sensitivity to broken rotational symmetry (in contrast to Table 3).

## Data Availability

The original contributions presented in this study are included in the article. Further inquiries can be directed to the corresponding author.
